# The Hospital Problem in London

**Published:** 1895-06-29

**Authors:** 


### The Hospital

An Institutional Journal of

The Medical Sciences and Hospital Administration, with

Vol. XVIII.—No. 457.] AN EXTRA NURSING SUPPLEMENT. [June 29, 1895.

### The Hospital Problem in London.

The Council of the Charing Cross Hospital have emphatically declared that the removal of that institution from its present site is unnecessary, inexpedient, and impracticable. This, in our opinion, is a wise decision on the part of Charing Cross Hospital, and we hope that it may lead to a conference with the King's College Hospital authorities, which will result in the amalgamation of the two medical schools, the purchase of the whole of the triangular site on a portion of which Charing Cross now stands, and the erection thereon of an adequate extension of the present hospital buildings. If these steps are taken they will of course involve the closing of King's College Hospital, and the transfer of its patients to the new buildings at Charing Cross. The sale of the King's College site, the reduction in administration expenditure, and the many economies and increased efficiency which might result cannot be exaggerated. Such a step would immeasurably increase the financial strength and influence of the amalgamated hospitals, would improve the schools of both institutions immensely, diminish the cost of administration, and prove a wise and prudent step, certain to be popular and successful. At any rate, the proposal to remove Charing Cross Hospital is now settled in the negative, and we congratulate the authorities upon their decision in the matter.

There remains the question of what is to be done, what is the best course to take in the matter of further hospital accommodation for South-east London. The Bishop of Rochester points out that London south of the Thames contains 1,600,000 inhabitants, that is, thrice the population of Liverpool and four times that of Leeds. The district grows bigger and poorer every year, and nobody seems to know or care. It has "two great general hospitals, St. Thomas's and Guy's. The work is beyond praise. Each needs and deserves increased support, and we are doing our utmost to obtain it. But where do they stand? On the very rim of our great circle, belonging almost as much to the north bank of the Thames as to the south. And behind him, in dreary uniformity, stretch some twenty square miles of dull common streets, most of them modern, nearly all of them poor—Walworth and Kennington, Camberwell and Peckham, Southwark and Bermondsey, Rotherhithe, Deptford, and the rest. If there be anywhere a region which needs a great general hospital in its midst it is this." This description of the Bishop of Rochester is precisely true and accurate in detail and in fact.

The House of Lords Committee, in view of the position as described by the Bishop, recommended the erection of a large general hospital at Camberwell, but the movement in South London languishes for want of money, although Leeds, with one-fourth the population, has a number of excellent hospitals which are adequately supported and excellently administered. The present position is a deadlock, owing to the negativing of the proposal to remove Charing Cross Hospital, and the failure of the inhabitants to supply the necessary money to found an entirely new institution.

It will be admitted by every thoughtful person who realises the meaning of the facts we have quoted from the Bishop's letter, that adequate hospital accommodation must be established and maintained in South London without further delay. How is this to be accomplished? By a scheme which will unite Guy's and St. Thomas's Hospitals in promoting the erection and maintenance of an outpost hospital at Camberwell, or on some favourable site near the centre of the district. As we have urged before, it is a wrong principle that the voluntary hospitals, which are, after all, nothing if they are not charities, should be so situated as to compel the majority of the patients who resort to them to incur loss and hardship because the sites on which they stand are situated miles away from the homes of the majority of the poor. We are strongly of opinion that the larger hospitals ought to follow the example of the Dreadnought Seamen's Hospital by planting outpost hospitals or receiving houses on convenient sites in the centre of the denser portions of the district which it serves. The branch or outpost hospital connected with the Dreadnought at the East and West India Docks has done good service to sick seamen, and its success should attract and fix the attention of the managers of every well-conducted general hospital in the metropolis. We are glad to hear active steps are being taken by those intimately associated with Guy's Hospital to establish an outpost hospital in South London either with or without the co-operation of St. Thomas's. Let the local committee place themselves in communication with the authorities of Guy's Hospital, when it should not be difficult to formulate a scheme which would be satisfactory to all parties, and at the same time secure adequate hospital provision under the happiest auspices in the district in question. We look forward to the time when we shall have in London a sufficient number of outpost hospitals for the reception of severe accident and urgent cases, working under the control of the larger general hospitals, where they can be at once attended to, and from which they can be brought in due course, if thought desirable, on a proper ambulance, to the parent institution.

